# Hyperosmotic treatment synergistically boost efficiency of cell-permeable peptides

**DOI:** 10.18632/oncotarget.9448

**Published:** 2016-05-18

**Authors:** Hu Wang, Ming Zhang, Fanhui Zeng, Changbai Liu

**Affiliations:** ^1^ The Institute of Cell Therapy, China Three Gorges University, Yichang, China; ^2^ Medical School, Department of Pathology, Biology and Immunology, China Three Gorges University, Yichang, China; ^3^ The Central Hospital of Enshi Tujia and Miao Autonomous Prefecture, Enshi, China; ^4^ Hubei Key Laboratory of Tumor Microenvironment and Immunotherapy, China Three Gorges University, Yichang, China

**Keywords:** cell penetrating peptide, hypertonic molecules, penetration, osmoprotectant

## Abstract

Therapeutics delivery into cells has been hurdled due to the barrier of cytoplasmic membrane. Although cell penetrating peptide (CPP) can potentially serve as an intracellular drug delivery vehicle, the application of CPP-based delivery is limited because the unsatisfactory delivery efficiency of CPP conjugated potent cargos is challenging their applications in present. Thus, the development of strategies for enhancing the penetrating efficiency of CPP would therefore urgent need to be explored to increase the scope of potential applications. We report here the effects of glucose, sucrose and manntiol (abbreviated as GSM) combination facilitating the penetration efficiency of CPP peptide alone or CPP-GFP (green fluorescence protein) conjugation in cultured cell lines or primary cells. Moreover, osmoprotectants glycerol and glycine supplementation help cells cope with the stress from GSM combination. Thus, our present study suggests that GSM combination in the presence of osmoprotectant can work as a new strategy for CPP penetration enhancement.

## INTRODUCTION

Macromolecules including nucleic acids (e.g. mRNA, siRNA, microRNA and lncRNA) and proteins (e.g. enzymes, cytokines and antibodies) have demonstrated great value as research tools and achieved widespread success as human therapeutics among the fastest growing classes of drugs [1, 2]. However, many powerful and potentially therapeutic proteins are typically not able to spontaneously enter into mammalian cells owing to the selectively permeable barrier of cellular membrane. To address this challenge, variety of macromolecule delivery approaches or reagents have been developed. Perhaps cell-penetrating peptide (CPP)-based delivery system is the most common and safe method for the introduction of exogenous macromolecule to transiently manipulate cell behavior without possibility of permanent genomic changes [3].

Examples of CPP also called protein transduction domains (PTD), because it can mediate the uptake of cargos that are covalently or non-covalently linked to them. Many CPPs have been discovered or applied during the past few decades, such as the TAT (human HIV-1 transactivator of transcription protein, residues 48–60), which have successfully delivered small molecules, peptides or proteins, nucleic acids, quantum dots (QDs), polysaccharides, liposomes and nanoparticles *in vitro* or *in vivo* [4]. Although TAT-mediated cargo delivery appears to work with almost all mammalian cells (from stem cells [5–8] to somatic cells [9, 10]) independent of tissue or organism types, insects and even plant cells, the unsatisfactory delivery efficiency of TAT or CPP conjugated potent cargos is challenging their applications at the clinical trial stage. Thus, the development of strategies for enhancing the penetrating efficiency of CPP would therefore urgent need to be explored to increase the scope of potential applications. Our group has discovered and successfully employed small molecule DMSO (dimethyl sulfoxide) [11] and BIT (1,2-benzisothiazolin-3-one) [12] to facilitate the penetrating efficiency of TAT or TAT fused conjugates for a range of cell types, however, the unknown mechanism of enhancement and their potential side effects in high concentration may affect their application in clinic.

Reports have shown that hypertonic molecules (e.g. sucrose and NaCl) treatment drives the highly efficient intracellular uptake of native proteins and other macromolecules into cells [13], and lysosomotrophic agents, such as chloroquine and sucrose have been used in CPP penetration to improve the efficiency of cargo delivery [11, 14], however, whether other agents such as glucose and manntiol as hypertonic molecules can enhance the penetration efficiency of CPP remain unknown. Here, we describe glucose and manntiol both enhance the penetrating efficiency of CPP, and we also found that glucose, sucrose and manntiol (abbreviated as GSM) can synergistically accelerate CPP entering into a wide variety of cell lines and primary cells. And osmoprotectants glycine and glycerol supplementation resulted in minimal effect on cell proliferation of different cell lines. Thus, we demonstrate that the system of CPP in hypertonic medium combined with osmoprotecants allows the highly efficient delivery of protein cargos.

## RESULTS

### The enhancement effect of GSM on TAT penetration

**Figure 1:**
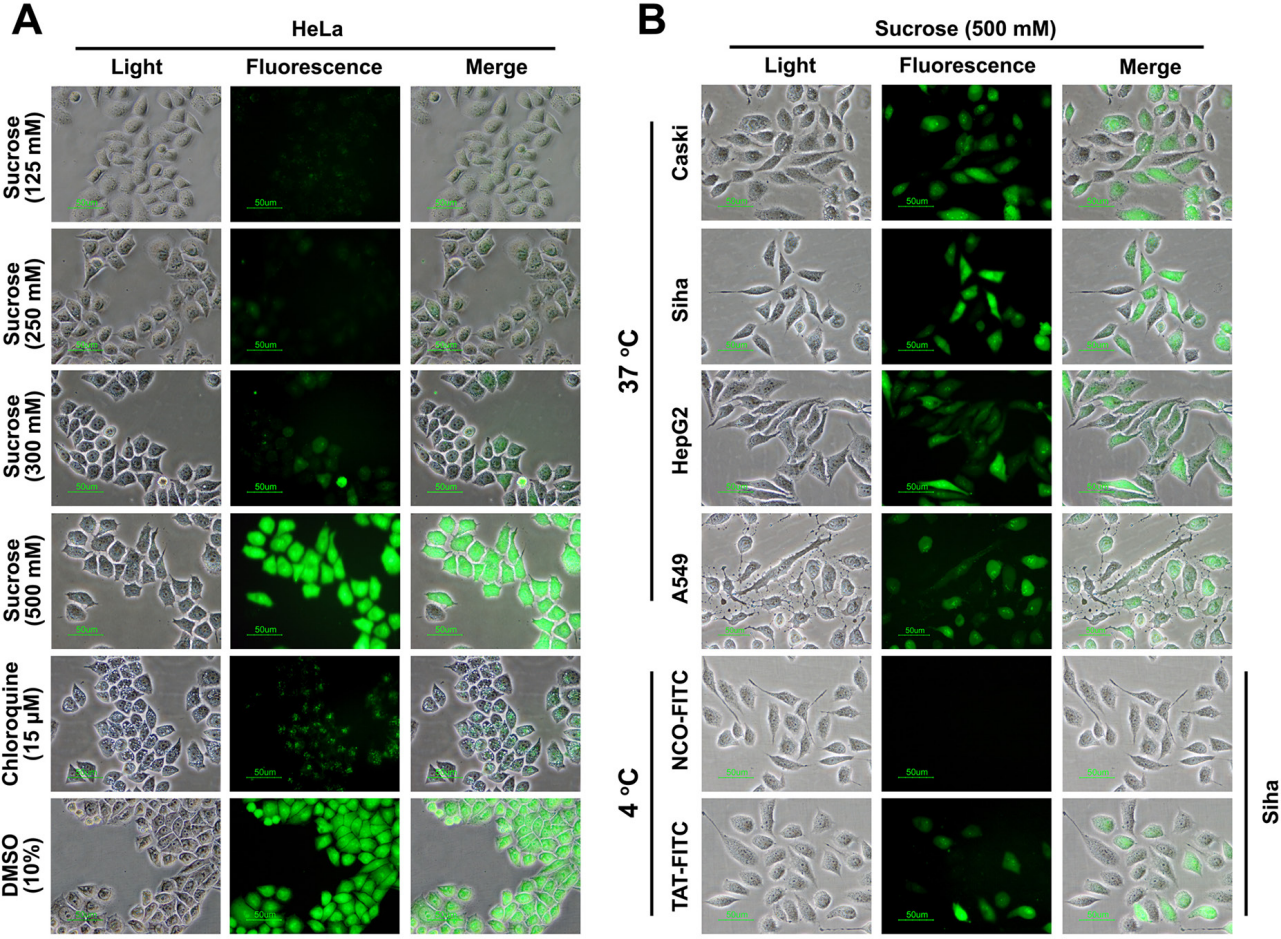
The penetrating efficiency of TAT can be enhanced by suitable concentration of sucrose, glucose as well as manntioll (**A**) Intracellular distribution of TAT-FITC detected by fluorescence microscopy. Chloroquine and DMSO treatment were used as control. (**B**) The subcellular distribution of TAT-FITC in different cell lines incubated with sucrose (500 μM) at 37°C or at 4°C.

**Figure 2:**
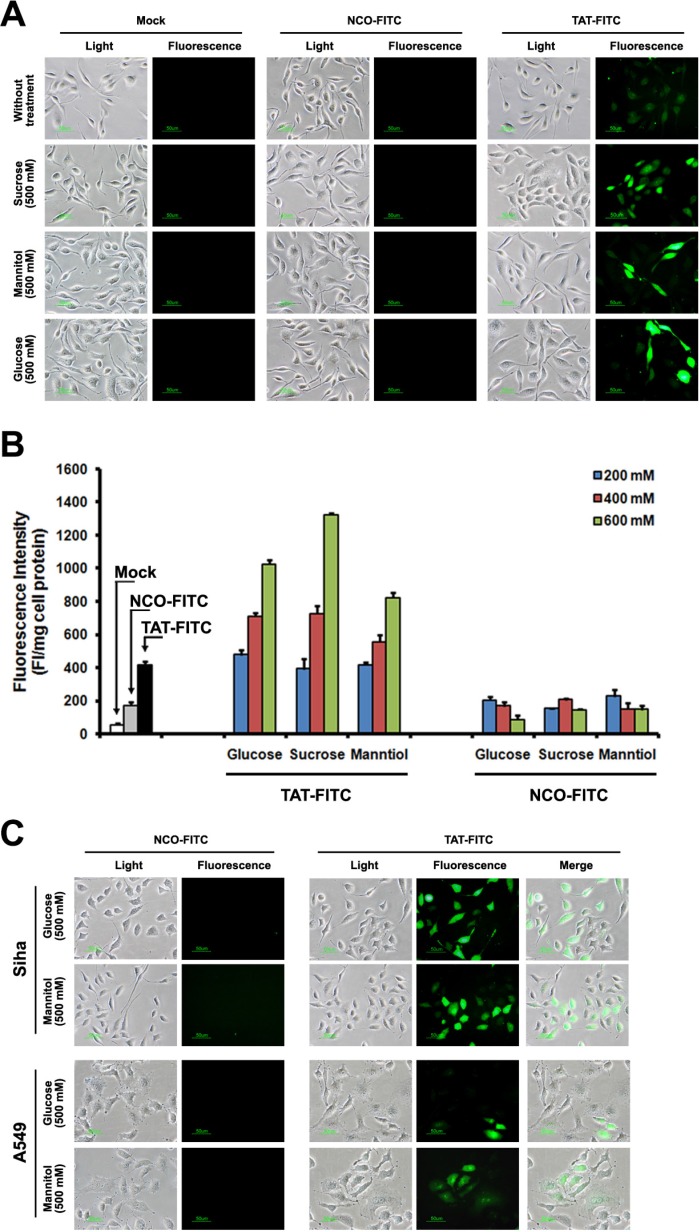
TAT-FITC uptaken by different cells treated with sucrose, glucose and manntiol (**A**) Caski cells were incubated with glucose, sucrose and manntiol (500 mM) for 1 h, and then treated with TAT-FITC for 1 h at 37°C. Fluorescence images were observed using fluorescence microscope. (**B**) Quantitative analysis of FITC-labeled TAT uptaken by Caski cells incubated with different concentration of glucose, sucrose and manntiol. (**C**) Fluorescence images of TAT-FITC uptaken by A549 and Siha cells incubated with glucose and manntiol.

A series of previous studies suggested that the suitable concentration of agents (chloroquine and sucrose) can promote the endosome-entrapped material release [15–17], thus, *in*-*vitro*-cultured cells incubated with sucrose can serve as an alternative strategy to enhance the drug delivery. To further examine the enhancement effect of sucrose on the penetration of TAT, we detected the intracellular distribution of TAT using fluorescence microscope, as shown in Figure 1A, TAT-FITC was well-distributed in the cytosol of HeLa cells incubated with sucrose (500 mM), it seems like that enhancement effect of chloroquine with different concentration is limited (Supplementary Figure S1), and we also detected intracellular distribution of TAT-FITC in different cells (Caski, A549, HepG2 and Siha) incubated with sucrose (500 mM) at 37°C for 1 h, as well as in Siha cells at 4°C (Figure 1B). These results suggest that suitable concentration of sucrose can be used to increase the penetration of TAT in different cell lines. Previous studies have shown that hypertonicity-inducing molecules (NaCl, RbCl, KCl and LiCl) could mediate protein delivery [13], and sucrose can enhance the DNA transfection as well [15], however, it is still unknown whether other hypertonicity-inducing agent including manntiol have similar effects. Therefore, we also examined the effect of glucose and manntiol on TAT penetration. In Figure 2A, the markedly TAT penetration enhancement was observed in Caski cells incubated with glucose and manntiol at 500 mM, and the dose ranging (from 200 to 600 mM) of glucose and manntiol experiments were also performed for quantitative analysis (Figure 2B), we discovered that TAT penetration enhancement in Caski cells was dependent on the concentration of glucose, sucrose and manntiol. Moreover, we explored the effect of different cell lines on TAT penetration in the presence of glucose and manntiol (Figure 2C), indicating that glucose and manntiol also can enhance the penetration of TAT uptake by different cell lines. In addition, galactose treatment also can partially enhance the penetration efficiency of TAT (Supplementary Figure S2A); however, the enhancement effect resulting from galactose treatment is lower than induction by manntiol (Supplementary Figure S2B).

### The combined effect of GSM on TAT penetration

**Figure 3:**
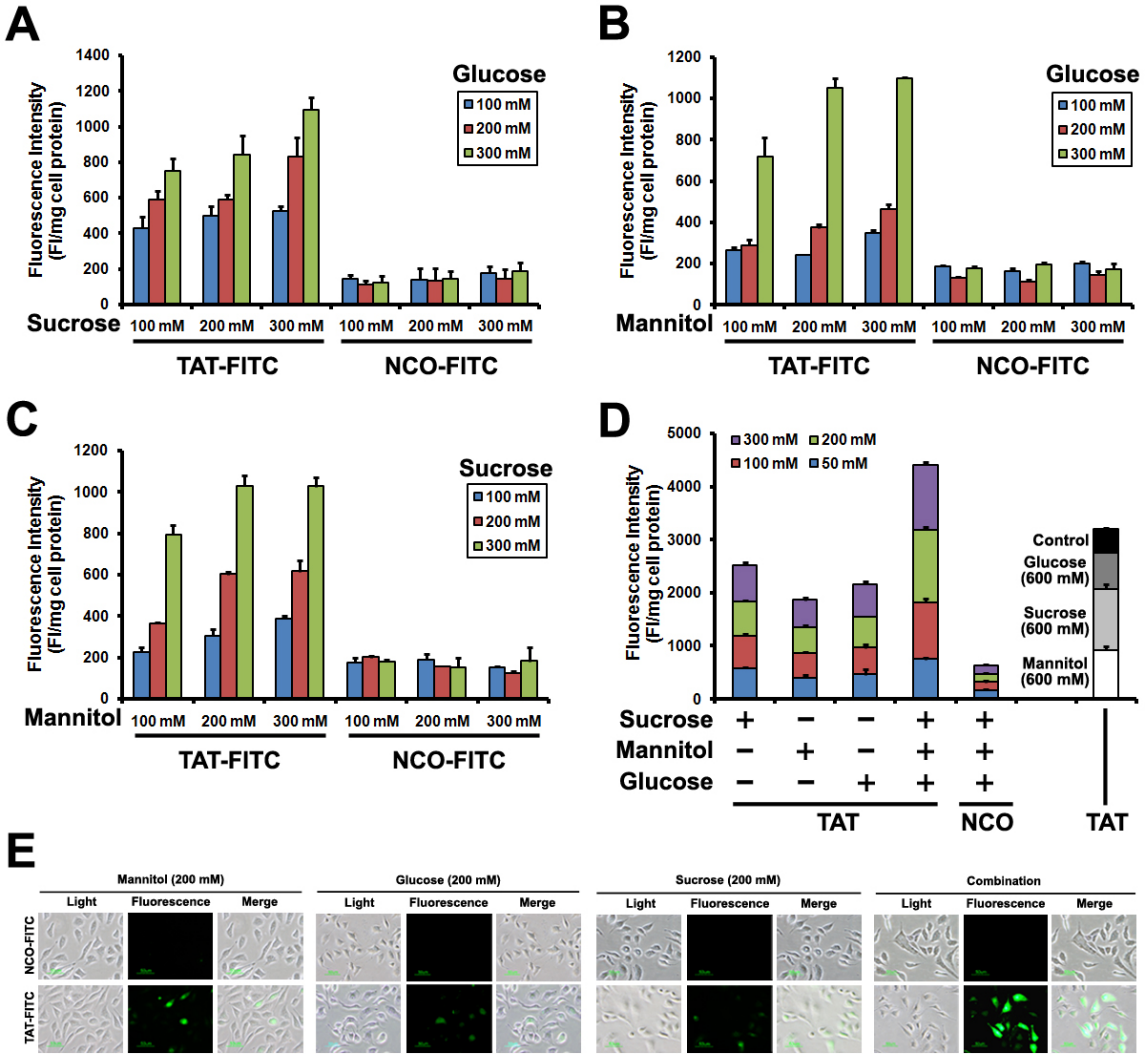
Quantitative analysis of Caski cells showing TAT-FITC penetration enhanced by co-incubation with glucose, sucrose and manntiol (**A**) Caski cells were co-incubated with glucose and sucrose (from 100 to 300 mM) for 1 h, and then treated with TAT-FITC for 1 h at 37°C. NCO-FITC group were used as negative control. (**B**) Caski cells were co-incubated with glucose and manntiol (from 100 to 300 mM) for 1 h, and then treated with TAT-FITC for 1 h at 37°C. (**C**) Caski cells were co-incubated with sucrose and manntiol (from 100 to 300 mM) for 1 h, and then treated with TAT-FITC for 1 h at 37°C. (**D**) Caski cells were co-incubated with glucose, sucrose and manntiol (from 50 to 300 mM) for 1 h, and then treated with TAT-FITC for 1 h at 37°C. TAT-FITC group incubated with glucose, sucrose and manntiol (600 mM) separately were used as control.

The above experiments suggest that TAT penetration efficiency was enhanced after glucose, sucrose and manntiol treatment. Next, we examined the TAT penetration efficiency in Caski cells incubated with glucose, sucrose and manntiol (abbreviated as GSM) combination. As shown in Figure 3A, TAT-FITC uptake by Caski cells was improved by glucose and sucrose combination, glucose and manntiol combination (Figure 3B) and sucrose and manntiol combination (Figure 3C). We also evaluated the combination effect of GSM. As expected, Figure 3D showed that GSM combination treatment lead to TAT-FITC uptake increasing as the concentration gradient, and Figure 3E shows an image of TAT penetration in Caski cells treated by GSM combination, and there was no difference between PBS washing and 0.025% trypsin washing method (Supplementary Figure S3). Thus, these data indicated co-incubation with GSM have combination effect.

### Protective of glycerol and glycine cotreatment

**Figure 4:**
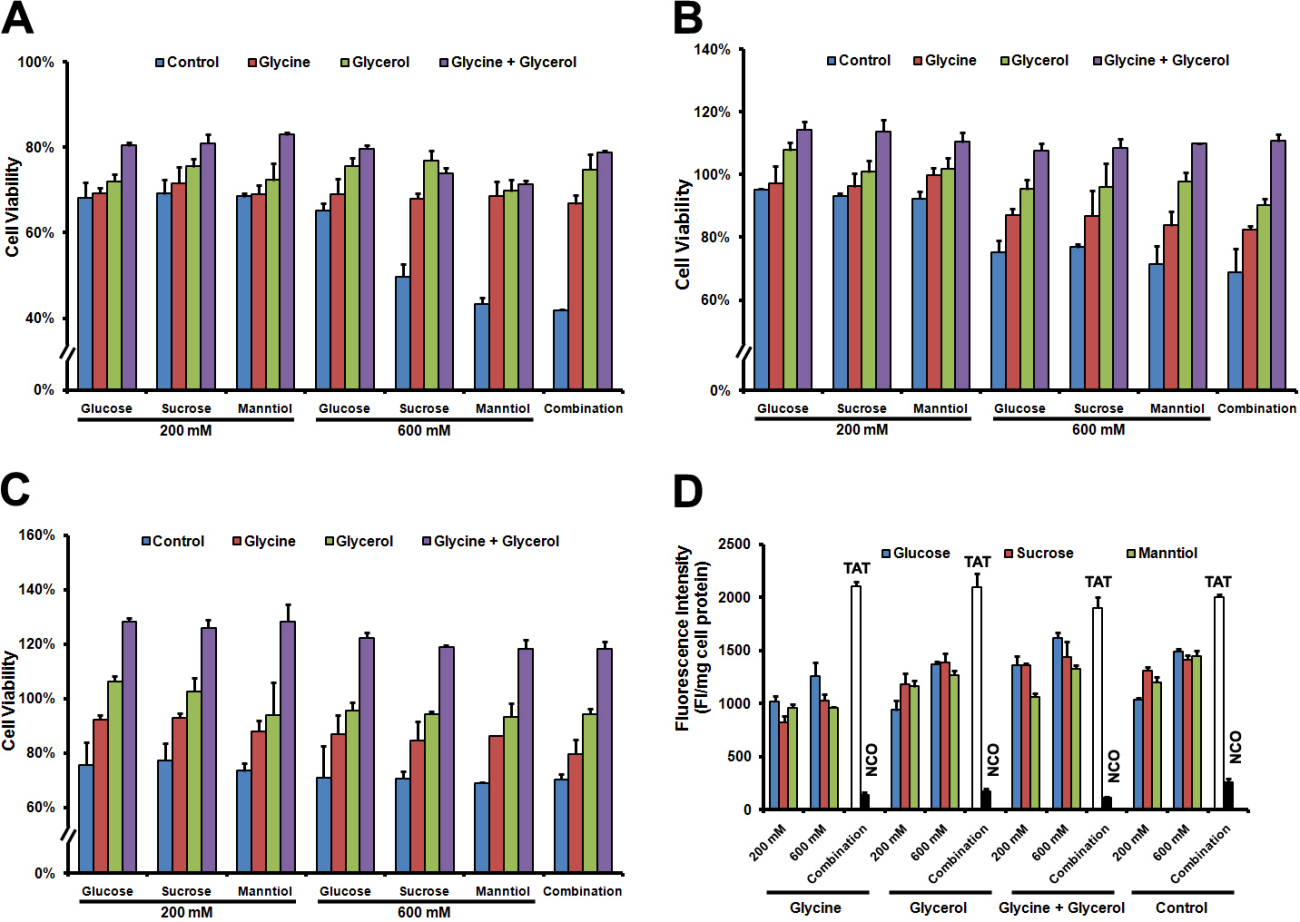
Cell viability of Caski cells rescued by glycerol and glycine combination (**A**) Caski cells were incubated glucose, sucrose and manntiol combination or separately as well as supplement with glycerol and glycine for 1 h at 37°C, discarding the supernatant of culture, washed with PBS 3 times, and then cultured for 24 h, cell viability was detected following the MTT protocol. (**B**) Cell viability was detected after 48 h culture with fresh medium; (**C**) Cell viability was detected after 72 h culture in fresh medium; (**D**) Quantitative analysis of FITC-labeled TAT uptaken by Caski cells incubated with glycerol and glycine combination or separately.

Although GSM combination promoted TAT-FITC penetration, cell survival may be affected by high concentration of sucrose and manntiol because they can act as afn osmotic [18]. However, recent report suggested that osmoprotectants such as glycerol and glycine can be used to help cells survive by balancing the osmotic effect [13]. Thus, we examined whether glycerol (30 mM) and glycine (15 mM) addition in media could prevent the hypertonicity induced the cell proliferation inhibition, without glycerol and glycine supplementation, Caski cells incubated with GSM separately or combination reduced the cell proliferation compared with control, however, glycerol and glycine addition during the GSM treatment can rescue the cell viability at 24 h (Figure 4A), 48 h (Figure 4B) as well as 72 h (Figure 4C), and still allowing the TAT penetration. We next investigated whether glycerol and glycine could potentially affect the TAT-FITC penetration ability as well, as shown in Figure 4D, TAT-FITC penetration efficiency was not observed in the presence of glycerol and glycine. These data demonstrate that glycerol and glycine only act as osmoprotectans but no significant penetration enhancing activity. Therefore, in the subsequent experiments, glycerol and glycine were added in the media for further analysis.

### Enhancement of TAT penetration by GSM combination in multiple cell types

**Figure 5:**
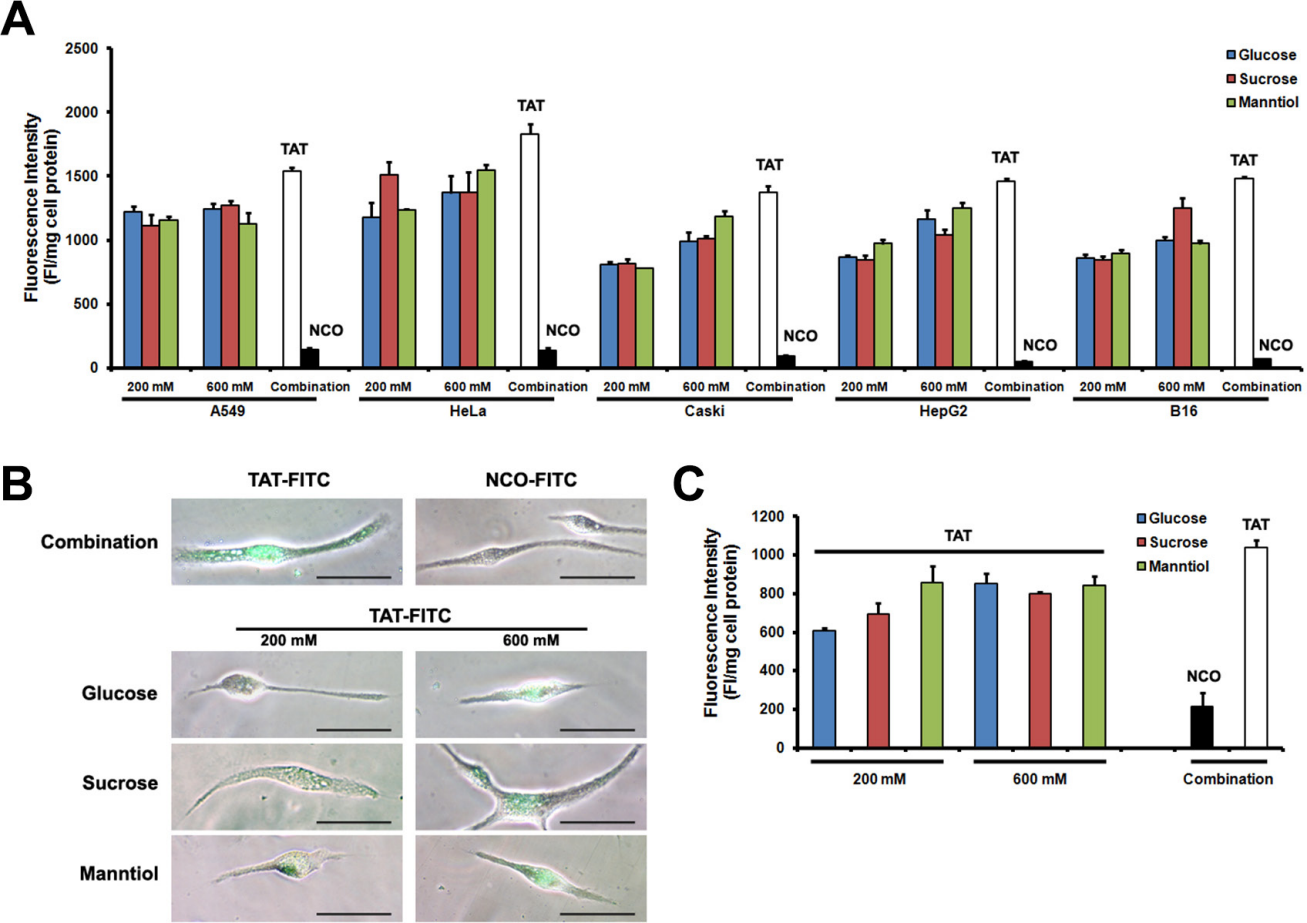
TAT-FITC uptaken by cultured cells and primary cells treated with glucose, sucrose and manntiol combination (**A**) Different cell lines were co-incubated with glucose, sucrose and manntiol (200 mM) for 1 h, and then treated with TAT-FITC for 1 h at 37°C. Quantitative analysis of FITC-labeled TAT uptaken was measured. (**B**) Fluorescence images of TAT-FITC uptaken by Sertoli cell incubated with glucose, sucrose and manntiol separately as well as combination. (**C**) Quantitative analysis of FITC-labeled TAT uptaken by Sertoli cell incubated with the combination of glucose, sucrose and manntiol.

To determine GSM combination efficiency in different cell types, we investigated GSM combination treatment in cultured cell lines as well as primary cultured cells. In Figure 5A, such combination treatment could potentially increase the FITC-labeled TAT uptake in different cancer cell lines *in vitro*. Figure 5B shows an image of TAT-FITC uptake in primary cultured Sertoli cells, and the fluorescence quantitative evaluation provide similar results to the microscopy (Figure 5C), demonstrating that GSM combination is still effective in both cell lines and primary cells.

### Enhancement of different CPPs penetration by GSM combination

**Figure 6:**
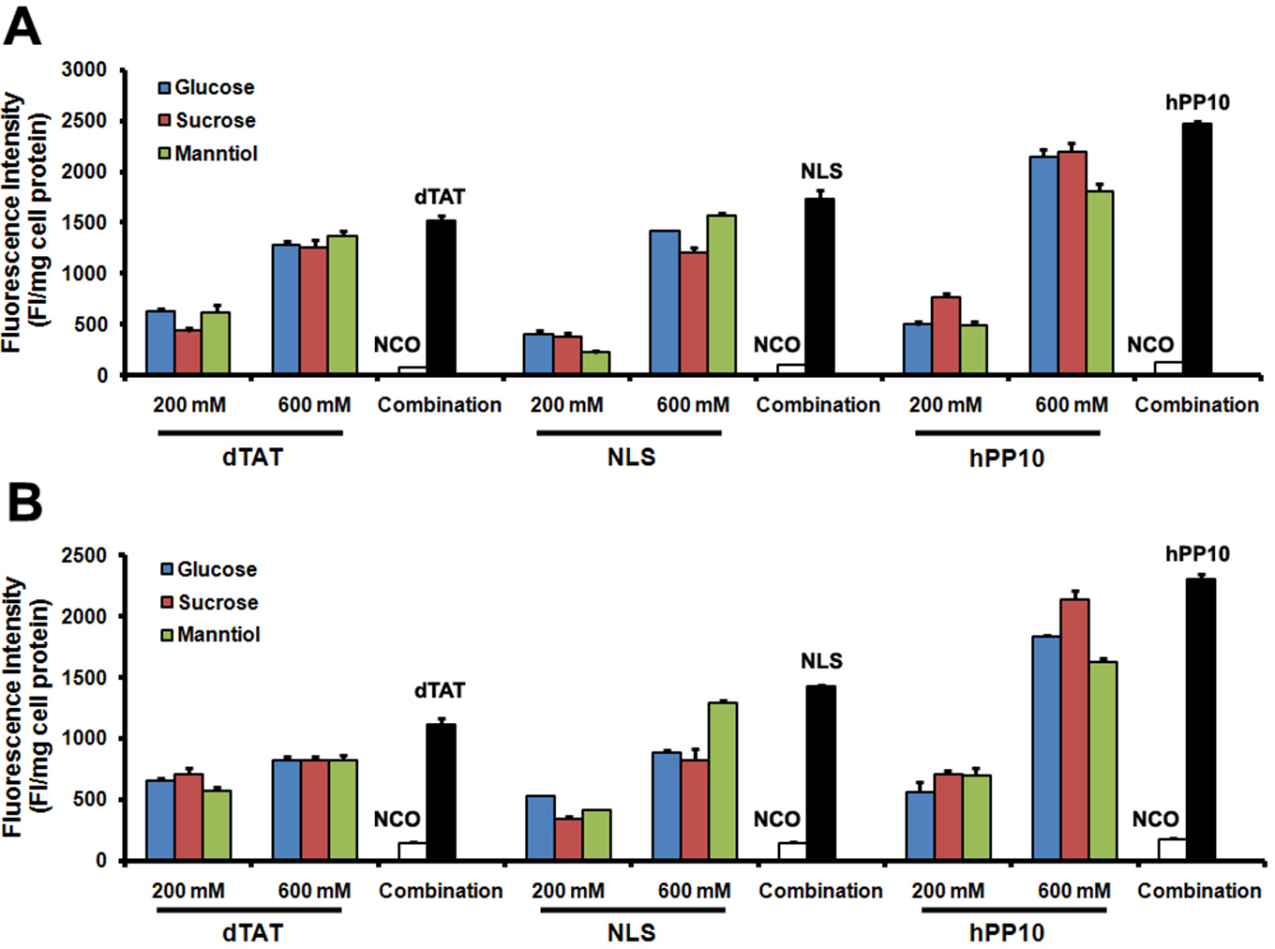
Different FITC-labeled peptides penetration in Caski and HepG2 cells (**A**) Quantitative analysis of dTAT-FITC, NLS-FITC and hPP10-FITC in Caski cells co-incubated with glucose, sucrose and manntiol. (**B**) Quantitative analysis of dTAT-FITC, NLS-FITC and hPP10-FITC in HepG2 cells co-incubated with glucose, sucrose and manntiol.

To explore whether GSM combination could enhance different kinds of CPPs including dTAT (tandem repeats of TAT), NLS and hPP10 (newly found human-derived CPP), Caski and HepG2 cells with TAT-FITC co-incubated with GSM were evaluated. As shown, GSM combination resulted in increased intracellular fluorescence intensity of TAT-FITC of Caski cells (Figure 6A) and HepG2 cells (Figure 6B). Altogether, these data demonstrate that GSM combination can facilitate the penetration efficiency of different kinds of CPPs.

### GSM combination does not affect TAT internalization pathway

**Figure 7:**
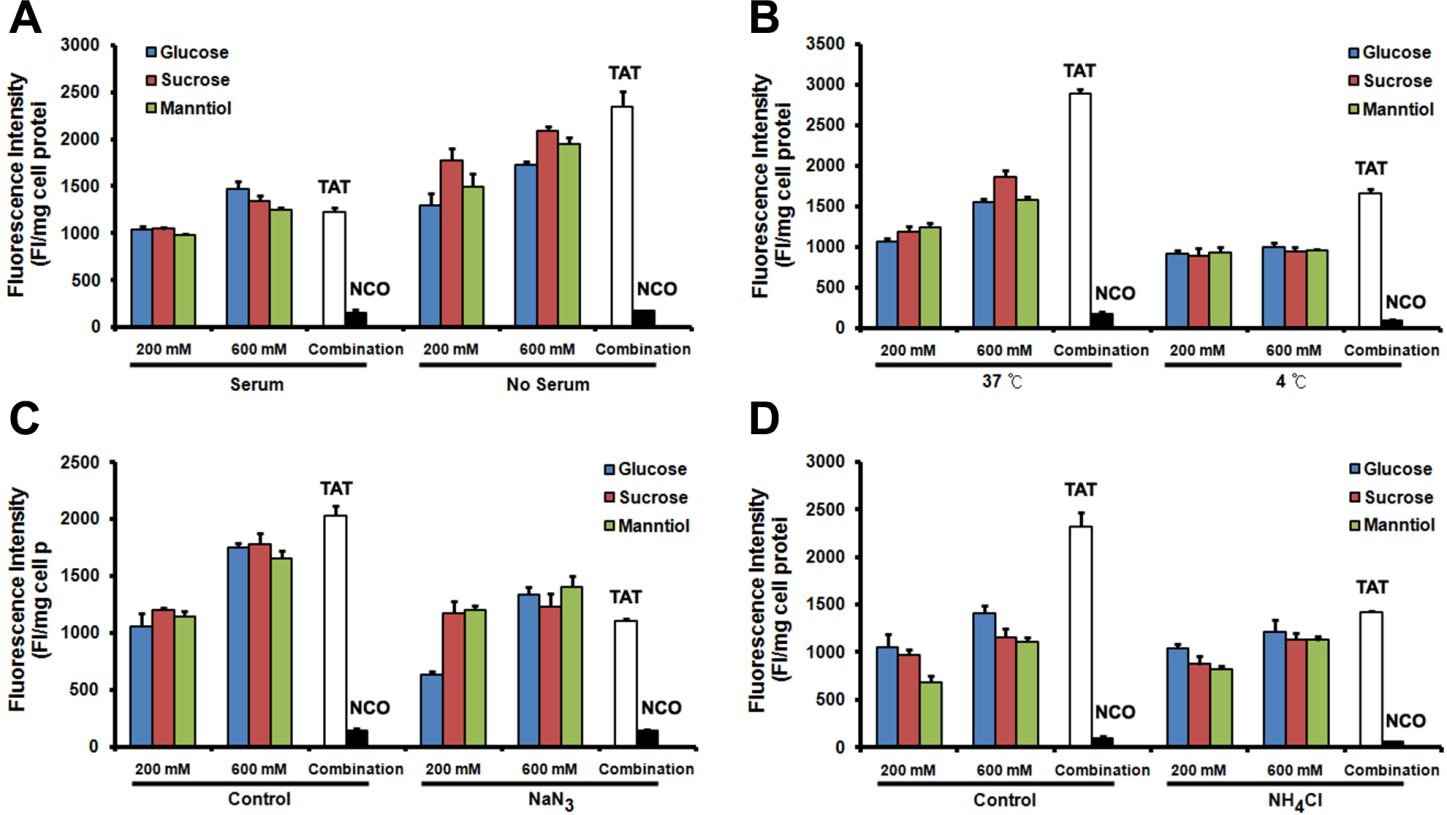
The mechanism of TAT-FITC penetration in Caski cell is not affected by glucose, sucrose and manntiol combination (**A**) Compared with none serum medium, serum contained medium incubation inhibit the combination effect of glucose, sucrose and manntiol. (**B**) Quantitative analysis of TAT-FITC in Caski cells co-incubated with glucose, sucrose and manntiol at 4 and 37°C. (**C**) Quantitative analysis of TAT-FITC in Caski cells co-incubated with glucose, sucrose and manntiol with or without the presence of sodium azide. (**D**) Quantitative analysis of TAT-FITC in Caski cells co-incubated with glucose, sucrose and manntiol with or without the presence of ammonium chloride.

Although the exact intracellular delivery mechanism of TAT remains elusive, we know that serum, temperature and endocytic inhibitors (such as sodium azide and ammonium chloride) affect the transduction level of TAT. To determine whether GSM combination affect the internalization pathway, we initially examined the TAT penetration efficiency in Caski cells cultured with or without 10% fetal bovine serum (FBS). TAT penetration efficiency remains decreased in FBS supplement group (Figure 7A) even in GSM combination. Next, we analyzed TAT penetration efficiency at different temperatures, the efficiency of TAT penetration decreased at lower temperatures (Figure 7B). In addition, we explored the effect of two inhibitors, including metabolic inhibitor NaN_3_ (Figure 7C) and lysosomotrophic neutralizing agent NH_4_Cl (Figure 7D) on TAT penetration efficiency. The intracellular delivery efficiency of TAT-FITC was significantly decreased in the presence of GSM combination treatment. Collectively, our results demonstrate that GSM combination does not affect TAT internalization pathway.

### TAT-GFP fusion protein uptake enhanced by GSM combination

**Figure 8:**
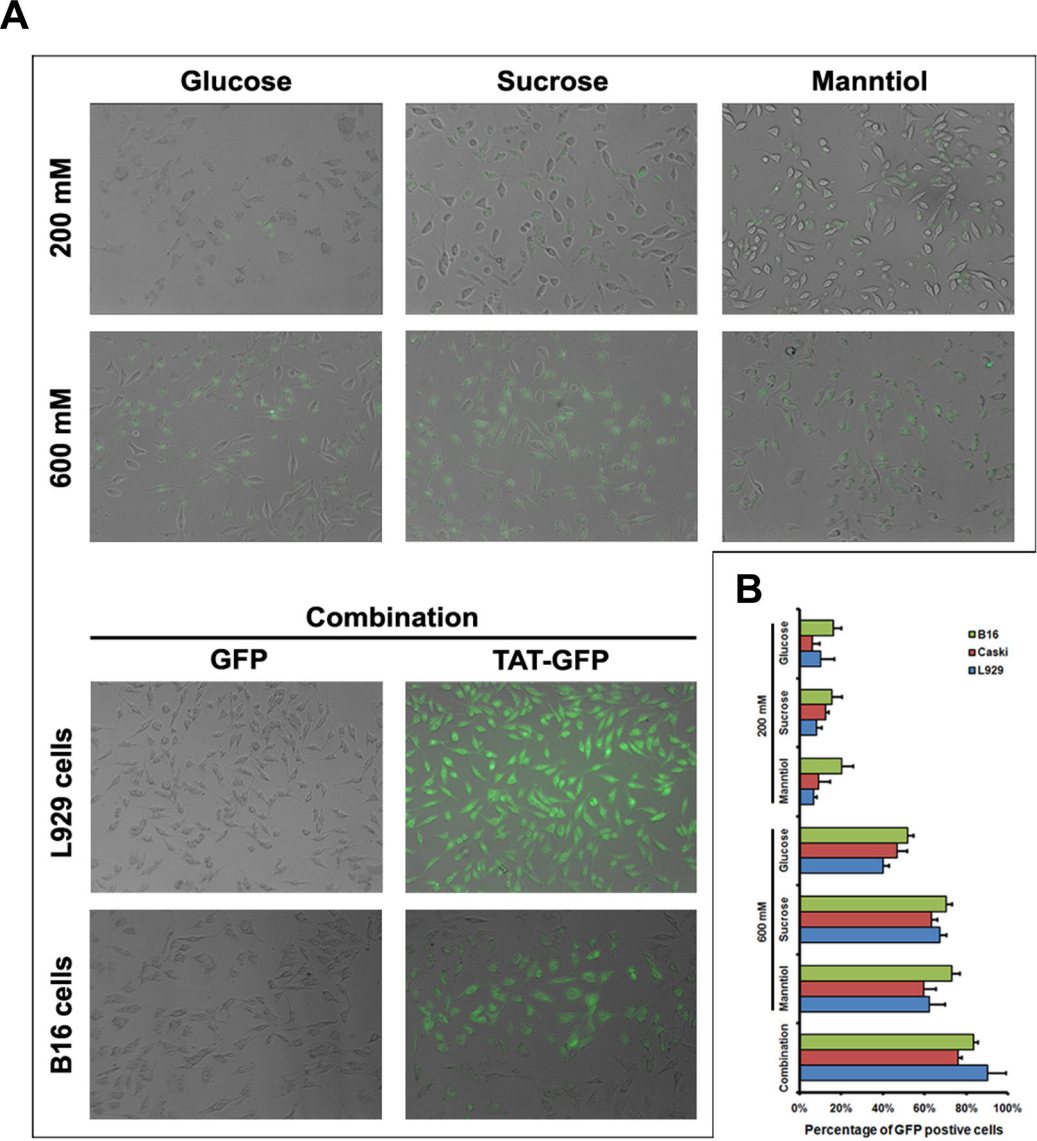
TAT-GFP fusion protein penetration enhanced by glucose, sucrose and manntiol combination (**A**) Fluorescence images of TAT-GFP uptaken by B16 and L929 cell incubated with glucose, sucrose and manntiol separately as well as combination. The top layer of image of transduced cells was L929 cell. (**B**) Quantitative analysis of TAT-GFP uptaken by Caski, B16 and L929 cell incubated with glucose, sucrose and manntiol separately as well as combination.

The above data demonstrate that GSM combination can be utilized to enhance the penetration efficiency of CPPs, however, it is extensively recognized that the uptake levels are related to the cargo sizes, and poor efficiency of CPPs fused to cargoes such as TAT-GFP crossing cells membrane were observed in different cell types (Supplementary Figure S4A), even in the presence of chloroquine (Supplementary Figure S4B) with different conditions. To further determine whether GSM combination treatment allows the penetration efficiency enhancement of TAT conjugated with fusion protein as well, we use TAT-GFP fusion protein as a direct detection system to quantify penetration efficiency. Following the incubation of GSM alone or combination for 1 h, fluorescence microscopy revealed that the enhancement of TAT-GFP penetration efficiency in GSM combination was more significant than the GSM alone in L929 and B16 cells (Figure 8A), similar result from fluorescence quantification of TAT-GFP uptake was measured in L929, B16 and Caski cells (Figure 8B). Thus, GSM combination treatment not only can enhance the penetration efficiency of TAT peptide, but also could potentially enhance the penetration of TAT-GFP fusion protein.

## DISCUSSION

Cell-penetrating peptides (CPP) are capable of delivering therapeutic cargo into live cells under physiological conditions and have been widely used for protein-based delivery [19]. Although CPP-based protein transduction appears to have enormous potential in the clinic, challenges for CPP-based delivery poses problems to the application due to the low effective cellular uptake efficiency. To our knowledge, osmotic gradient across the cell membrane could be caused by sucrose treatment, and hypertonic treatment using sucrose was used to promote endosome release of endosome-entrapped material [15, 16]. Here, we have introduced that other hypertonic molecules including manntiol as well as sucrose could be used to enhance the penetration efficiency of CPP, and GSM combination in certain concentration (200 mM) facilitate the CPP alone or CPP conjugated cargo penetration highly in cultured cell lines and primary cells compared with GSM treatment separately. Moreover, glycerol and glycine co-treatment could alleviate the proliferation inhibition of GSM combination. Indeed, hypertonic solutions containing sucrose, glucose and manntiol were used in the treatment of cerebral edema for osmotic therapy [18].

Although the previous study suggested that hypertonic shock can be used to enhance the cargo delivery, from the present study, hypertonic treatment without recovery period still can facilitate the CPP-based cargo delivery. Next, glucose, sucrose as well as manntiol can be modified by some groups so as to improve the stability of these molecules which will be very helpful for *in vivo* usage. Previous studies have reported that sucrose can be used to help the CPP-cargo release from the endosome, thus, we conclude that glucose, sucrose and manntiol may also through the endosome release promotion to enhance the penetration of CPP. Secondly, ion channels are very important for cell survival as well as for small molecules delivery, more important, different ion channels' activity may vary in different cells, whether the different enhancement effect of GSM combination due to different cells is still unknown, and whether the ion channels play a role in the enhancement by GSM combination treatment is still unknown. However, the non-CPP conjugated cargo releasing are much lower than CPP, thus leading to the GSM combination enhance the CPP-cargo delivery higher than non-CPP based delivery.

GSM combination can significantly improve the penetration efficiency of CPP in the presence of glycerol and glycine, although the exact mechanism of enhancement still needs to be investigated in the further research. Our present research may lay a foundation to CPP-based bio-therapy for brain tumor patient with symptoms of intracranial hypertension.

## MATERIALS AND METHODS

### Reagents and drugs

NaN_3_, heparin and NH_4_Cl were purchased from Shandong Wanbang Biochem Co. Ltd (Shandong, China). Chloroquine diphosphate salt was provided by HongJing Chem Co. Ltd (Hubei, China). Trypsin was supplied by Sigma-Aldrich (USA) and dimethyl sulfoxide (DMSO) was obtained from Sigma.

### Cell lines and cell culture

HeLa, Siha and Caski (human cervical cancer cell line), A549 (human nonsmall cell lung cancer cell line), B16 (mouse melanoma cell line), HepG2 (human hepatocellular carcinoma cell line) were maintained in our laboratory. All cells were maintained in RPMI 1640 medium supplemented with 10% FBS and 1% penicillin/streptomycin, and were cultured at 37°C in a humidified atmosphere containing 5% CO_2_.

### Sertoli cell isolation, purification and culture

All mice were got from government approved animal facility at China Three Gorges University. All experimental protocols were reviewed and approved by the China Three Gorges University Institutional Animal Care and Use Committee. All efforts were done to minimise animal suffering, the mice were deeply anesthetized with ether and then sacrificed by cervical dislocation to collect the testes. Sertoli cells were isolated from CD1 mice following the protocol previously described [20–22]. As Sertoli cells from mature male mouse cannot be cultured very well, 4 testes from 10 days postnatal males were collected and placed in PBS, decapsulated with scissors, cut into small fragments and digested in 0.05% trypsin for 10 min shaking at 37°C in water bath to remove the interstitium. The resultant seminiferous tubule fragments were collected and then washed with PBS followed by further 0.05% trypsin digestion under the same conditions for 10 min. The disaggregated samples were filtered using a 70 μm filter and then centrifuged at 800 g for 10 min. The mixed population of testes cells obtained were resuspended in culture medium (DMEM supplement with 10% FBS plus 100 U/ml penicillin and 100 μg/ml streptomycin), then plated on gelatin coated dishes and incubated for 1 h at 37°C in a humidified atmosphere of 5% CO2. After the incubation, non-adhering cells were removed by washing twice with culture medium. The attached cells are mainly Sertoli cells which will be cultured for 7–10 days and then used for penetration experiment following the protocol described below.

### FITC-labeled peptides synthesis and fusion protein expression

Peptides TAT (sequence: YGRKKRRQRRRK), NCO (a nonsense peptide, sequence: KALGISYGRKK), NLS (sequence: RKDRRGGEMMKQKRQRE), dTAT (tandem repeat of TAT) and hPP10 (sequence: KIPLPRFKLKCIFCKKRRKR) labeled with FITC were synthesized from SBS Genetech (Beijing, China). These peptides were purified using reversed phase analytical HPLC (more than 99% purity), diluted to 500 mM and stored at −20°C for further use. TAT-GFP and GFP protein were prepared following the protocol described [11, 12], fusion proteins were expressed in the BL21 (DE3) strain of *E. coli*, and 6-His-tagged target proteins were purified by affinity chromatography using Ni-NTA resins.

### Peptide internalization

### Fluorescent microscopy

TAT-FITC treated cells were observed under a fluorescence microscope. HeLa, Siha, Caski, HepG2, A549 or B16 cells (density: 5 × 10^5^ cells per well) were seeded into 24-well-plates (Greiner, Germany) and cultivated in RPMI-1640 medium for 24 h. Before the 10 μM FITC-labeled peptides addition, cells were washed with phosphate-buffered saline (0.1 M PBS, pH 7.4) for 3 times. After internalization for 1 h at 37°C, cells were washed 3 times with PBS, and then observed under fluorescence microscope (Nikon, Japan) using a bandpass filter (detects FITC). In some experiments, cell was pretreated with 5% (v/v %) of DMSO in serum-free medium.

### Fluorescent quantitation

Intracellular fluorescence intensity of cells can be quantified using quantitative analysis by Multimode Spectrophotometry (Tecan 2000, Mannedorf, Switzerland). Briefly, after peptide incubation procedure shown above, cells were washed 3 times with PBS, lysed by 0.1 M NaOH at room temperature, and then centrifuged at 14000 g for 5 min. The fluorescence intensity of the supernatant was measured at the wavelength of 494/518 nm in a Multimode Microplate Reader. The fluorescence of cellular uptake is expressed as fluorescence intensity per mg of total cellular protein (protein concentration were determined using Bradford protein assay). All experiments were repeated at least three times and always performed in triplicate.

### Cell viability analysis

MTT assay was used to assess cell viability. Briefly, cells (4 × 10^4^ cells) were seeded in one well of 96-well plates and cultivated in an RPMI-1640 medium for 24 h. Cells were washed with PBS and then adding different concentrations of supplements with a serum-free medium, incubated for 2 h at 37°C and washed with PBS twice, then incubated with fresh medium containing serum for 24 h. Then, 20 μl of MTT (5.5 mg/ml) in serum-free media was added directly to each well and incubated for 4 h at 37°C. Removing the supernatant and then adding 100 μl of DMSO, and incubated at 37°C for another 30 min before quantifying the absorbance at 550 nm.

### Statistical analysis

All experiments were performed at least three times and all results are expressed as means ± standard deviation (SD). Statistical significance between groups was calculated using SPSS software. A student's *t*-test was used for data analysis and *p* value < 0.05 was taken as the level of statistically significant.

## SUPPLEMENTARY MATERIALS FIGURES


