# Supplementary material for: Hyperosmotic treatment synergistically boost efficiency of cell-permeable peptides
Source: Oncotarget. 2016 May 18;7(46):74648–57. doi: 10.18632/oncotarget.9448 (PMC5342692; doi:10.18632/oncotarget.9448)
Supplement: Supplementary file 1 [file oncotarget-07-74648-s001.pdf]

Hyperosmotic treatment synergistically boost efficiency of cell-permeable peptides

Supplementary Materials

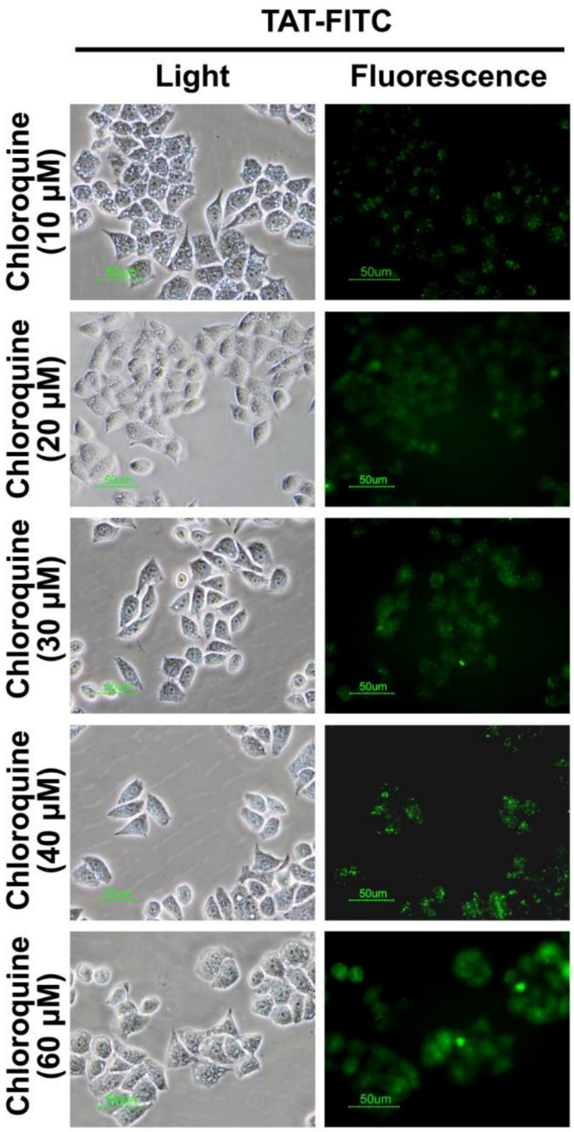

Supplementary Figure S1: Intracellular distribution of TAT-FITC uptaken by Caski cell incubated with different concentration of chloroquine.

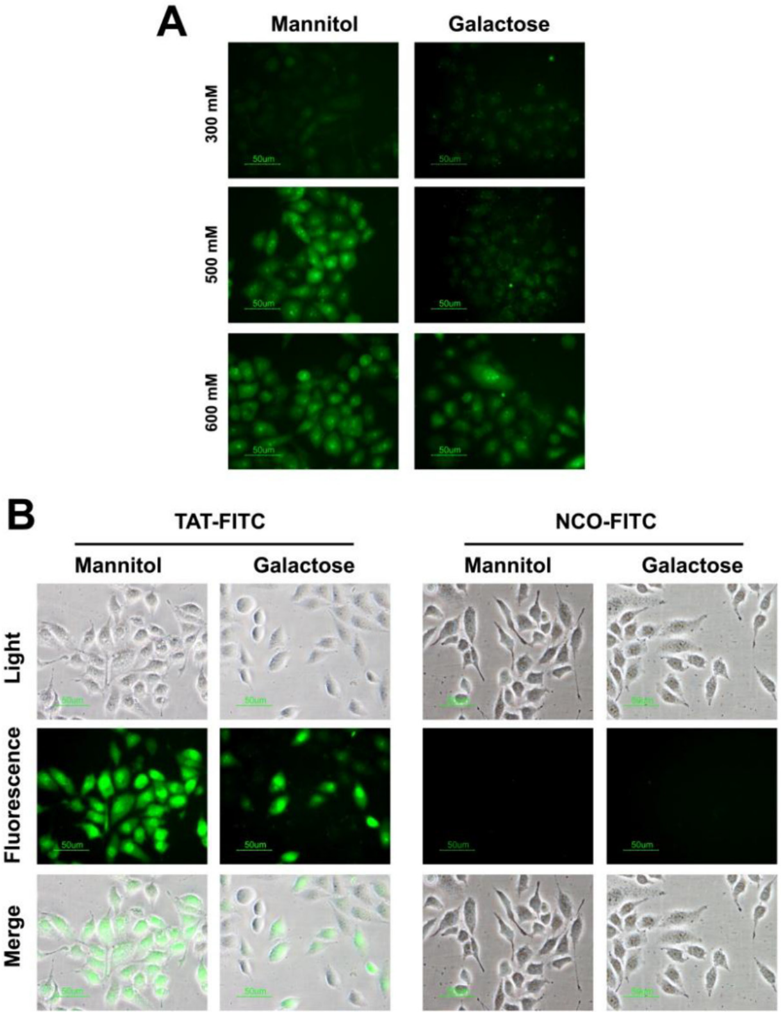

Supplementary Figure S2: Intracellular distribution of TAT-FITC uptaken by Caski cells incubated with galactose. (**A**) Fluorescence images of TAT-FITC uptaken by Caski cells incubated with different concentration of galactose and manntiol. (**B**) Fluorescence images of TAT-FITC uptaken by Siha cells incubated with galactose (500 mM) and manntiol (500 mM).

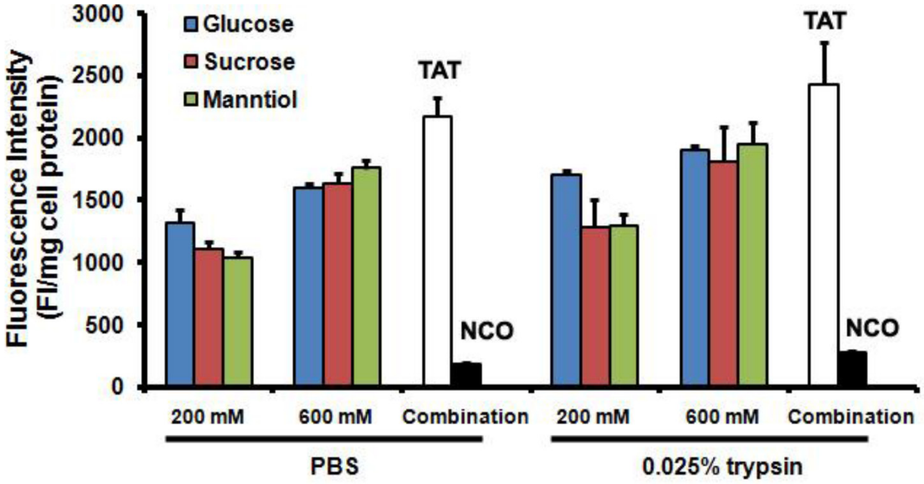

Supplementary Figure S3: Different washing methods have no effects on the combination of glucose, sucrose and manntiol treatment.

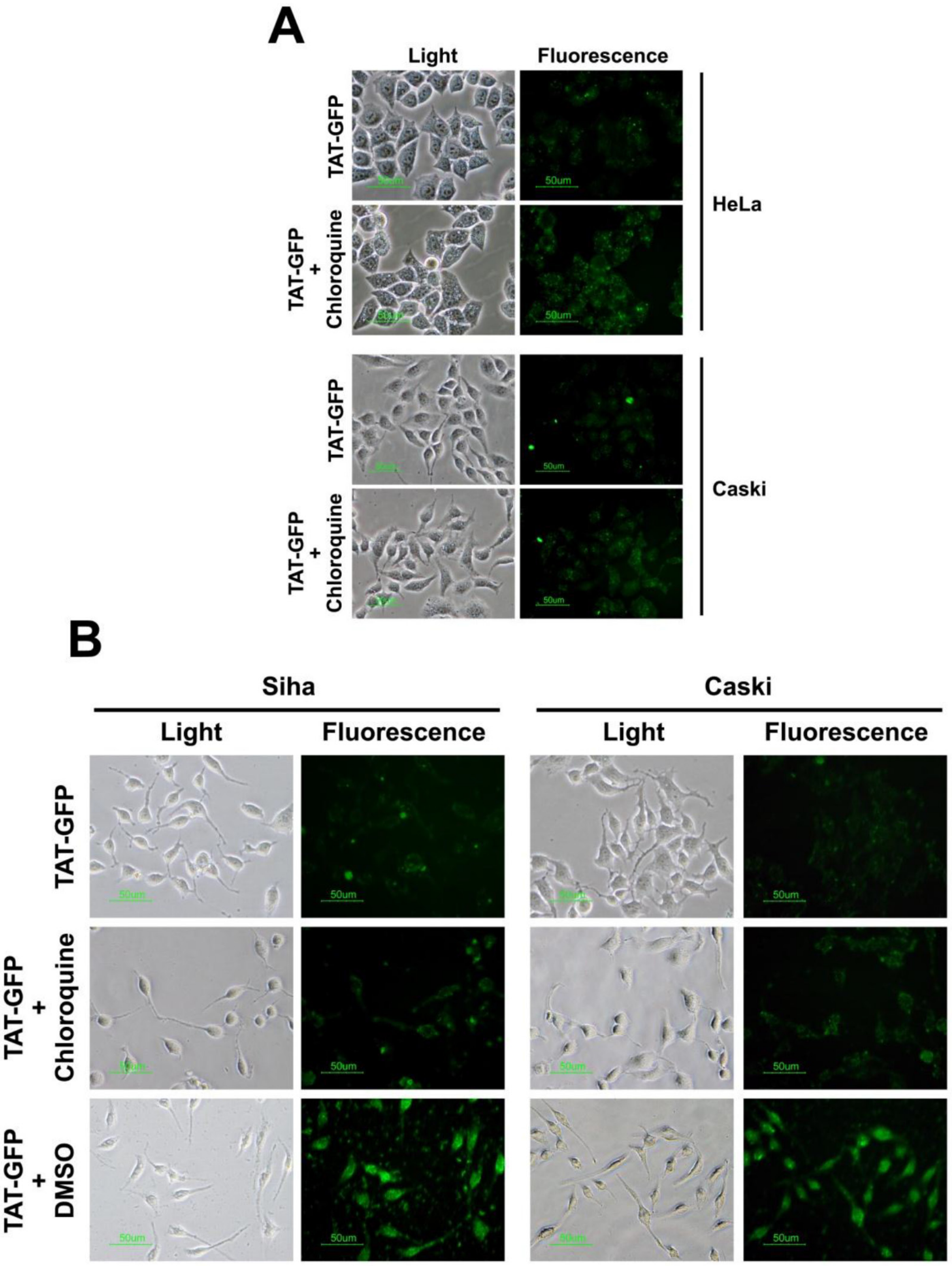

Supplementary Figure S4: Intracellular distribution of TAT-GFP uptaken by different cells incubated with or without chloroquine. (**A**) Fluorescence images of TAT-GFP uptaken by Caski and HeLa cells incubated with or without chloroquine at 37°C. (**B**) Fluorescence images of TAT-GFP uptaken by Caski and Siha cells at 37°C in the presence of chloroquine or DMSO.
